# Supplementary material for: Non-communicable diseases and resistant tuberculosis, a growing burden among people living with HIV in Eastern Kenya
Source: PLOS Glob Public Health. 2025 Jun 20;5(6):e0004212. doi: 10.1371/journal.pgph.0004212 (PMC12180655; doi:10.1371/journal.pgph.0004212)
Supplement: Attachment — Submitted filename: Peer reviewers comments.docx [file pgph.0004212.s002.docx]

| Line | Quote from manuscript | Comment |
| --- | --- | --- |
| 22-24 | “Countries with the highest rates of people living with HIV (PLWH) also have a high prevalence of non-communicable diseases (NCDs), including type 2 diabetes (T2D) and hypertension (HPT).” | Consider restructuring this sentence. Though it is grammatically correct, it is not as fluent. |
| 27 | “TB and rifampicin resistance” | Please look into this part and consider "Rifampicin resistance...." and remove "TB and..." |
| 49-50 | “**Keywords:** Non-communicable disease, sensitive and multidrug-resistant Tuberculosis, Burden, Predictors, People living with HIV, Kenya | consider adding "Rifampicin resistant" |
| 57 | “94% achieving **virological suppression**. Increased life expectancy among PLWH has become… | consider including the number of copies.  Example: "... virological suppression of less than 200 copies." |
| 59 | **“… T**uberculosis (TB) as well as non-communicable diseases (NCDs) such as obesity,” | lowercase "T" |
| 62 | “…**Tuberculosis** is still a major cause of | The abbreviation has already been introduced above. Just write "TB" |
| 82 | “…**vice versa; a reverse** association may exist, such that HPT may lead to an increased risk of | This part does not read well. It does not flow nicely for a reader. Rather "...vice versa. However, a reverse..." |
| 90 | “..to active disease in the general population is **affected** by factors such as **cigarette** smoking,..” | Affected: Consider using the word "influenced" instead of "affected".  Cigarette: verify this from this source, as "tobacco smoking" would be more ideal than "cigarette" smoking. This because cigarette is just packaging of tobacco, whereas scientifically, the main risk factor is tobacco smoking... whether cigar, cigarette, hookah/hubbly bubbly etc. consider verify from the source or paraphrase. |
| 99-100 | **Study design**  “This study was undertaken among PLWH from 10th June 2021 99 to 9th June 2022 in Nyambene Sub-County Hospital in Eastern Kenya.” | While this is important, is not study design, but the setting or area and duration. Was it a cohort/prospective cohort, cross-sectional, RCT or case control? |
| 111 | “…of 19.8% [15], a total of 280 eligible PLWH were recruited with 15% added to cover for lost” | "recruited" this means they were approached, screened whether they meet eligibility criteria and obtaining consent to screen.  Enrolled: Means the consent was obtained successfully and the participant was included in the study.  rather say "recruited and enrolled" or simply enrolled. |
|  |  |  |

Overall, the statistical analysis is done well, findings are well presented, and the manuscript is well written and reads well. Only minor corrections required in the **background** and **methodology.**
