# Supplementary material for: Non-communicable diseases and resistant tuberculosis, a growing burden among people living with HIV in Eastern Kenya
Source: PLOS Glob Public Health. 2025 Jun 20;5(6):e0004212. doi: 10.1371/journal.pgph.0004212 (PMC12180655; doi:10.1371/journal.pgph.0004212)
Supplement: Attachment — Submitted filename: Response to Reviewers.docx [file pgph.0004212.s004.docx]

12^th^ May, 2025

Lei Gao
Academic Editor
PLOS Global Public Health

Dear Editor,

**Re: Response to reviewers’ comments PGPH-D-25-00036**

Thank you for creating time to review our manuscript. Non-communicable diseases and resistant tuberculosis, a growing burden among people living with HIV in Eastern Kenya

Journal Requirements:

**Comment 1**. Your current Financial Disclosure states, “The study was supported by student Loan”. However, your funding information on the submission form indicates that you did not receive funding. Please indicate by return email the full and correct funding information for your study and confirm the order in which funding contributions should appear. Please be sure to indicate whether the funders played any role in the study design, data collection and analysis, decision to publish, or preparation of the manuscript.

**Response**: The study was supported by student Loan. The funders **did not** contribute in any way to the study design, data collection and analysis, decision to publish, or preparation of the manuscript.

**Comments 2**: We do not publish any copyright or trademark symbols that usually accompany proprietary names, eg (R), (C), or TM  (e.g. next to drug or reagent names). Please remove all instances of trademark/copyright symbols throughout the text, including ®, ™ on page 13, 11, 12.

**Response:** All the copyright or trademark symbols have been removed from the manuscript

**Comment 3.** Please review your reference list to ensure that it is complete and correct. If you have cited papers that have been retracted, please include the rationale for doing so in the manuscript text, or remove these references and replace them with relevant current references. Any changes to the reference list should be mentioned in the rebuttal letter that accompanies your revised manuscript. If you need to cite a retracted article, indicate the article’s retracted status in the References list and also include a citation and full reference for the retraction notice.

**Response**: The reference list is complete and correct

**Reviewer's Responses to Questions**

**Comments to the Author**

1. Does this manuscript meet PLOS Global Public Health’s [publication criteria](https://journals.plos.org/globalpublichealth/s/criteria-for-publication)? Is the manuscript technically sound, and do the data support the conclusions? The manuscript must describe methodologically and ethically rigorous research with conclusions that are appropriately drawn based on the data presented.

Reviewer #1: Yes

Reviewer #2: Yes

**Response**: Thank you, reviewers, for finding that this manuscript meets the PLOS Global Public Health’s publication criteria

**Comment 2.** Has the statistical analysis been performed appropriately and rigorously?

Reviewer #1: Yes

Reviewer #2: Yes

**Response**: Thank you, reviewers, for finding the statistical analysis performed appropriately and rigorously

**Comment 3**. Have the authors made all data underlying the findings in their manuscript fully available (please refer to the Data Availability Statement at the start of the manuscript PDF file)?

Reviewer #1: Yes

Reviewer #2: Yes

**Response**: Thank you, reviewers, for this comment. The study data is fully available for review

**Comment 5. Review Comments to the Author**

Reviewer #1: This is important work and I appreciate the focus on the overlap.

I would focus more in the introduction and conclusion and some analysis on 1) diabetes being a risk for TB (one of the leading risks, replacing malnutrition and HIV in some locations) and a risk for more severe outcomes
https://www.who.int/news/item/07-02-2025-who-operational-handbook-on-tb-and-comorbidities--diabetes#:~:text=According%20to%20the%202024%20WHO,a%20high%20prevalence%20of%20TB.
https://pmc.ncbi.nlm.nih.gov/articles/PMC11342417/
https://pmc.ncbi.nlm.nih.gov/articles/PMC10662654/

and HIV as a risk for HTN, which is more complicated and may be mediated by weight gain with ARVs, but also persistent HIV is a risk for Hypertension and it can additionally be mediated by the virus' impact on the cardiovascular and renal system directly, which leads to HTN risks.
https://pmc.ncbi.nlm.nih.gov/articles/PMC7877548/
https://academic.oup.com/ofid/article/12/Supplement_1/ofae631.773/7988171?login=false
https://pmc.ncbi.nlm.nih.gov/articles/PMC11886560/
https://pmc.ncbi.nlm.nih.gov/articles/PMC6002926/
Additionally TB, especially if advanced, can lead to HTN, if

**Response**: Thank you for this comment, while the main focus of this manuscript was too highlight the burden and predictors of NCDs and TB among PLWH in Eastern Kenya, the study has attempted to show the interaction of diabetes as a risk factor for TB has been shown in several section of the manuscript, such as: abstract line 43-45, Introduction lines 84-89 and in the discussion line 326 to 359. HIV as a risk for HTN has been highlighted throughout the manuscript. Introduction lines 85 to 88, discussion lines 319-323. Further, two separate manuscripts describing the interaction of diabetes as a risk for TB and HIV as a risk for HTN are underway. This way, we will provide significant details of these two important diseases and their interactions.

**Comment**: I would use this focus to inform further statistical analyses. There are different approaches which can be used to improve the statistical analysis including methods of considering the likely causal relationships here. You could consider expanding the multivariable analysis to include all comorbidity combinations instead of just HIV/TB, which would provide more comprehensive insights into these complex disease interactions. The analysis would benefit from including interaction terms (e.g., Diabetes × TB severity, HIV × Hypertension) to examine whether these conditions have multiplicative rather than just additive effects. Stratifying analyses by HIV status and ART duration would better assess how diabetes affects TB risk differently in various patient subgroups. Time-to-event models, whenever possible, would provide a more dynamic analysis of how diabetes duration affects TB risk and how ART duration influences hypertension incidence. Applying propensity score matching would help balance baseline characteristics between comparison groups, minimizing selection bias and strengthening causal inferences from this observational study.

**Response**: Thank you for this comment, just as summarized in table 1 and 2, in the multivariate models, as explained in the section covering Factors associated with the comorbidity of TB and Lifestyle diseases among PLWH, specifically Lines 260 to 266, multivariate analysis were done for all the various possible comorbidity combinations including HIV/TB, HIV/T2D, HIV/HPT, HIV/MDR TB, HIV/TB/T2D, HIV/TB/HPT, HIV/T2D/HPT, and HIV/TB/T2D/HPT and baseline variables, only HIV/TB comorbidity was found significant.

More advanced statistical analyses, such as Time-to-event models, propensity score matching, among others, are being applied to the two specific manuscripts in preparation.

**Comment**: Would reword this "6.4% HIV/T2D/HPT coinfection" and "3.2% PLWH coinfected with TB/HPT" and "quadruple coinfection of NCDs and TB" and "Triple coinfections included 18 (6.4%) HIV/T2D/HPT,
37 9 (3.2%) HIV/TB/T2D, and 9 (3.2%) HIV/TB/HPT, with a quadruple coinfection of
38 HIV/TB/T2D/HPT among 4 (1.4%) participants." etc Hypertension and diabetes are not infections.

**Response**: Thank you for this observation. For cases where Hypertension and diabetes are reported, we have replaced co-infection with comorbidities in the abstract section, lines 35 to 38, as follows: The majority of the participants, 179 (63.9%), were HIV mono-infected. The dual comorbidities reported included 58 (20.7%) HIV/TB, 42 (15%) HIV/T2D, and 33 (11.8%) HIV/HPT. Triple comorbidities included 18 (6.4%) HIV/T2D/HPT, 9 (3.2%) HIV/TB/T2D, and 9 (3.2%) HIV/TB/HPT, with a quadruple comorbidity of HIV/TB/T2D/HPT among 4 (1.4%) participants

**Comment**: Would capitalize differently: coronavirus (coViD-19) pandemic,

Response: Thank you for this comment, the suggestions have been adopted as shown in line 67, the coronavirus (COVID-19) pandemic

Comment Reviewer #2: Overall, the statistical analysis is done well, findings are well presented, and the manuscript is well written and reads well. Only minor corrections required in the background and methodology. Thank you!

**Response**: Thank you for this observation. The minor corrections in the attachment have been implemented

| Line | Quote from manuscript | Reviewers Comment | Response |
| --- | --- | --- | --- |
| 22-24 | “Countries with the highest rates of people living with HIV (PLWH) also have a high prevalence of non-communicable diseases (NCDs), including type 2 diabetes (T2D) and hypertension (HPT).” | Consider restructuring this sentence. Though it is grammatically correct, it is not as fluent. |  |
| 27 | “TB and rifampicin resistance” | Please look into this part and consider "Rifampicin resistance...." and remove "TB and..." | Thank you for this comment. The word TB removed |
| 49-50 | “**Keywords:** Non-communicable disease, sensitive and multidrug-resistant Tuberculosis, Burden, Predictors, People living with HIV, Kenya | consider adding "Rifampicin resistant" | Thank you for this comment: Key words made more specific…. sensitive and Rifampicin-resistant Tuberculosis, Burden, Predictors, People living with HIV, Kenya |
| 57 | “94% achieving **virological suppression**. Increased life expectancy among PLWH has become… | consider including the number of copies.  Example: "... virological suppression of less than 200 copies." | Thank you for this comment. Viral copies of (< 200 copies/mL) added. |
| 59 | **“… T**uberculosis (TB) as well as non-communicable diseases (NCDs) such as obesity,” | lowercase "T" | Thank you for this comment. T changed to lower case |
| 62 | “…**Tuberculosis** is still a major cause of | The abbreviation has already been introduced above. Just write "TB" | Thank you for this comment. **Tuberculosis** replaced with TB |
| 82 | “…**vice versa; a reverse** association may exist, such that HPT may lead to an increased risk of | This part does not read well. It does not flow nicely for a reader. Rather "...vice versa. However, a reverse..." | Thank you for this comment. The wording **vice versa deleted …** development of HPT, and a reverse association… |
| 90 | “..to active disease in the general population is **affected** by factors such as **cigarette** smoking,..” | Affected: Consider using the word "influenced" instead of "affected".  Cigarette: verify this from this source, as "tobacco smoking" would be more ideal than "cigarette" smoking. This because cigarette is just packaging of tobacco, whereas scientifically, the main risk factor is tobacco smoking... whether cigar, cigarette, hookah/hubbly bubbly etc. consider verify from the source or paraphrase. | Thank you for this observation. Affected replaced by influenced on the same line.  Cigarette smoking replaced with tobacco smoking throughout this manuscript |
| 99-100 | **Study design**  “This study was undertaken among PLWH from 10th June 2021 99 to 9th June 2022 in Nyambene Sub-County Hospital in Eastern Kenya.” | While this is important, is not study design, but the setting or area and duration. Was it a cohort/prospective cohort, cross-sectional, RCT or case control? | Thank you for this observation The study design clarified. This cross-sectional study was undertaken among ….. |
| 111 | “…of 19.8% [15], a total of 280 eligible PLWH were recruited with 15% added to cover for lost” | "recruited" this means they were approached, screened whether they meet eligibility criteria and obtaining consent to screen.  Enrolled: Means the consent was obtained successfully and the participant was included in the study.  rather say "recruited and enrolled" or simply enrolled. | Thank you for this comment. The term recruited replaced by enrolled…of 19.8% [15], a total of 280 eligible PLWH were enrolled with 15% added to cover for lost to follow up. |

Thank you for finding time to provide important reviews to our manuscript. We appreciate your time and look forward for a positive response.

Kind regards,

**Dr. Musa Otieno Ngayo**

Centre for Microbiology Research

KEMRI
